# Development of a Novel Peptide Nucleic Acid Probe for the Detection of *Legionella* spp. in Water Samples

**DOI:** 10.3390/microorganisms10071409

**Published:** 2022-07-13

**Authors:** Montserrat Nácher-Vázquez, Ana Barbosa, Inês Armelim, Andreia Sofia Azevedo, Gonçalo Nieto Almeida, Cristina Pizarro, Nuno Filipe Azevedo, Carina Almeida, Laura Cerqueira

**Affiliations:** 1 LEPABE—Laboratory for Process Engineering, Environment, Biotechnology and Energy, Faculty of Engineering, University of Porto, Rua Dr. Roberto Frias, 4200-465 Porto, Portugal; montserratnv@gmail.com (M.N.-V.); anabarbosa@fe.up.pt (A.B.); ines.mr.armelim@gmail.com (I.A.); asazevedo@fe.up.pt (A.S.A.); nazevedo@fe.up.pt (N.F.A.); carina.almeida@iniav.pt (C.A.); 2 INIAV, IP—National Institute for Agrarian and Veterinary Research, Vairão, 4485-655 Vila Do Conde, Portugal; goncalo.almeida@iniav.pt; 3 ALiCE—Associate Laboratory in Chemical Engineering, Faculty of Engineering, University of Porto, Rua Dr. Roberto Frias, 4200-465 Porto, Portugal; 4 i3S—Instituto de Investigação e Inovação em Saúde, Universidade do Porto, 4200-135 Porto, Portugal; 5 IPATIMUP—Instituto de Patologia e Imunologia Molecular, Universidade do Porto, 4200-135 Porto, Portugal; 6 INSA—National Institute of Health Doutor Ricardo Jorge, Rua Alexandre Herculano 321, 4000-055 Porto, Portugal; cristina.pizarro@insa.min-saude.pt; 7 Centre of Biological Engineering (CEB), University of Minho, Campus de Gualtar, 4710-057 Braga, Portugal

**Keywords:** fluorescence in situ hybridization, *Legionella* sp., peptide nucleic acid (PNA), 16S rRNA gene, waterborne detection

## Abstract

*Legionella* are opportunistic intracellular pathogens that are found throughout the environment. The *Legionella* contamination of water systems represents a serious social problem that can lead to severe diseases, which can manifest as both Pontiac fever and Legionnaires’ disease (LD) infections. Fluorescence in situ hybridization using nucleic acid mimic probes (NAM-FISH) is a powerful and versatile technique for bacterial detection. By optimizing a peptide nucleic acid (PNA) sequence based on fluorescently selective binding to specific bacterial rRNA sequences, we established a new PNA-FISH method that has been successfully designed for the specific detection of the genus *Legionella*. The LEG22 PNA probe has shown great theoretical performance, presenting 99.9% specificity and 96.9% sensitivity. We also demonstrated that the PNA-FISH approach presents a good signal-to-noise ratio when applied in artificially contaminated water samples directly on filtration membranes or after cells elution. For water samples with higher turbidity (from cooling tower water systems), there is still the need for further method optimization in order to detect cellular contents and to overcome interferents’ autofluorescence, which hinders probe signal visualization. Nevertheless, this work shows that the PNA-FISH approach could be a promising alternative for the rapid (3–4 h) and accurate detection of *Legionella*.

## 1. Introduction

*Legionella* is a Gram-negative bacteria, ubiquitously found in natural freshwater, as well as in man-made water systems, such as cooling towers, hospitals or whirlpool spas [1]. From the 60 *Legionella* species, more than 20 are known to infect humans, causing serious illnesses, such as Legionnaires’ disease (LD) and Pontiac fever [2]. From these, *Legionella pneumophila* is responsible for about 97% of all outbreaks, and the other 3% is mainly due to non-*L. pneumophila* species, such as *L. longbeachae*, *L. micdadei* and *L. bozemanii* [3]. Upon the inhalation of aerosols, *Legionella* has the ability to colonize and replicate in the human alveolar macrophages, triggering the disease [4]. Closer surveillance and the use of methods for diagnosis in water management are critical for effectively reducing the public risk caused by *Legionella*. Moreover, according to official authorities, taking a more active role in minimization of *Legionella* in water supply systems, including the implementation of stronger programs for monitoring and testing, will be vital in decreasing disease outbreaks [5]. Despite the excellent developments in the area of identification, the current technologies do not fully answer the needs of industrial entities for *Legionella* monitoring.

The use of the International Organization for Standardization (ISO) 11731:2017 is recommended for the isolation and determination of *Legionella* in water samples (potable, industrial, waste and natural) using the gold standard culture method [6]. However, this method is a laborious and time-consuming procedure (taking more than a week to provide the results), often with low sensitivity, especially in samples of complex matrices, as in biofilms, and does not allow for the detection of viable but non-cultivable cells (VBNC) [7]. Therefore, it is important to develop culture-independent detection methods for microbial monitoring. PCR-based methods (ISO 12869:2019) [8] have been proposed for monitoring *Legionella* in environmental systems, but the interpretation of results from environmental samples remains difficult, leading to an overestimation of the actual health risk [9,10]. These molecular methods can detect the number of genome units (GU) per liter, but there is still a need for correspondence with the number of colony forming units (CFU), which is usually lower, probably due to VBNC cells’ presence [11]. Research has been conducted in order to develop, optimize and validate new molecular methods, and fluorescence in situ hybridization (FISH) has been shown to be a promising technology [12,13,14,15,16].

FISH is a visual, culture-independent molecular technique that allows for the rapid detection and quantification of microorganisms [17]. This method is typically based on specific binding of fluorescently labeled nucleic acid probes, generally DNA molecules, to particular rRNA sequences of microorganisms [18]. However, the development of synthetic nucleic acid mimics (NAMs), such as peptide nucleic acid (PNA), has been shown to provide an improved hybridization performance compared to DNA probes [19,20]. PNA chemical structure allows for the bases to be positioned at equivalent spatial arrangement as DNA, which allows for the hybridization with DNA/RNA molecules to occur, obeying the Watson–Crick base-pairing rules [19]. Moreover, they present a stronger affinity to the target sequences and subsequently higher accuracy, mainly explained by the fact that these molecules are neutrally charged, reducing the electrostatic repulsion that occurs between negatively charged DNA/DNA or RNA/RNA duplexes [21,22]. PNA-FISH has been used for the rapid detection (approximately 3 h) of several relevant microorganisms, such as bacteria [23,24,25,26,27], yeasts [28,29] and filamentous fungi [13,30,31]. Regarding *Legionella*, only one *Legionella* spp. (PLEG200) probe and one *L. pneumophila* PNA probe were identified [32] in the literature. These probes were based on pre-existing DNA probes targeting a low-binding-affinity site on the 16S rRNA. In this work, we attempted to design a new PNA-FISH method for the specific detection of the genus *Legionella*, targeting a distinctive conserved region of 16S rRNA. In addition, preliminary assays, using the PNA-FISH method, were tested in artificial water samples to ensure that this method could be successfully applied to environmental samples.

## 2. Materials and Methods

### 2.1. Strain and Growth Conditions

The bacterial strains used in this study are indicated in Table 1. A total of 17 strains of different *Legionella* species and 19 non-*Legionella* bacteria were tested. The bacteria collection was supplied by Laboratório de Microbiologia do Departamento de Saúde Ambiental Porto do Instituto Nacional de Saúde Doutor Ricardo Jorge (INSA-DSA ASMIP) and by Prof. Manuel Simões (LEPABE) [33]. All *Legionella* strains were grown on buffered charcoal yeast extract agar (BCYE) at 37 °C for 2 to 4 days. Non-*Legionella* strains were grown on tryptic soy agar (TSA) (3% (*w*/*v*) tryptic soy broth and 1.5% agar) at 37 °C for 24 h, except *Pseudomonas fluorescens*, which was incubated at 30 °C.

### 2.2. In Silico PNA Probe Design for the Specific Detection of Legionella

**Figure 1:**
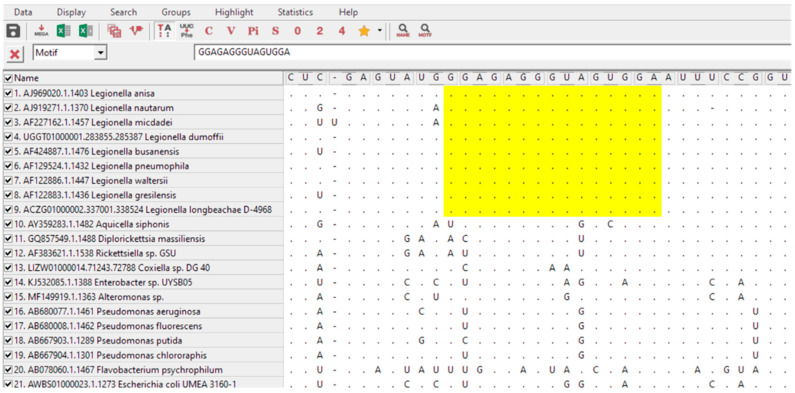
Partial alignment of 16S rRNA sequences for *Legionella* probe selection using ClustalW from MEGA-X software. Yellow shadow shows the selected region that matched all *Legionella* sequences but did not match the nontarget sequences. The sequence shown corresponds with LEG22 complementary reverse sequence.

For the design of a specific *Legionella* spp. probe, an approach using available alignment programs coupled with the 16S rRNA databases was used according to the methodology described by Teixeira et al. (2021) [34]. From these, a group of 9 target *Legionella* and 27 non-target sequences were selected from the ARB Silva database (https://www.arb-silva.de/; accessed on May 2020). Only sequences with a length above 1200 bp, high sequence quality (>90%), high alignment quality (>90%) and high pintail quality (>90%) were selected. Regions of interest were identified with MEGA-X and aligned with ClustalW (https://www.megasoftware.net/; last accession on June 2020) (Figure 1). High GC percentage and low number of consecutive self-complementary nucleotides were also criteria to consider in sequence selection.

The theoretical specificity and sensitivity were calculated using the Probe Match tool on the Ribosomal Database Project (RDP-II) (http://rdp.cme.msu.edu; last accession on June 2020) as previously described by Almeida et al. (2010) [35]. Specificity was calculated as nLs/(TnL) × 100, where nLs stands for the number of non-*Legionella* sequences that did not align with the probe, and TnL is the total of non-*Legionella* sequences examined. Sensitivity was calculated as Ls/(TLs) × 100, where Ls stands for the number of *Legionella* sequences detected by the probe, and TLs is the total number of *Legionella* strains present in the databases. The selected sequence was synthesized (Eurogentec, Seraing, Belgium). The N terminus was labeled with AlexaFluor^®^ 488 via a double 8-amino-3,6-dioxaoctanoic acid (AEEA) linker. Additionally, the theoretical evaluation of a previously published *Legionella* spp. PNA probe [32] was performed to compare the theoretical accuracy of the selected probe.

### 2.3. Hybridization Conditions

**Figure 2:**
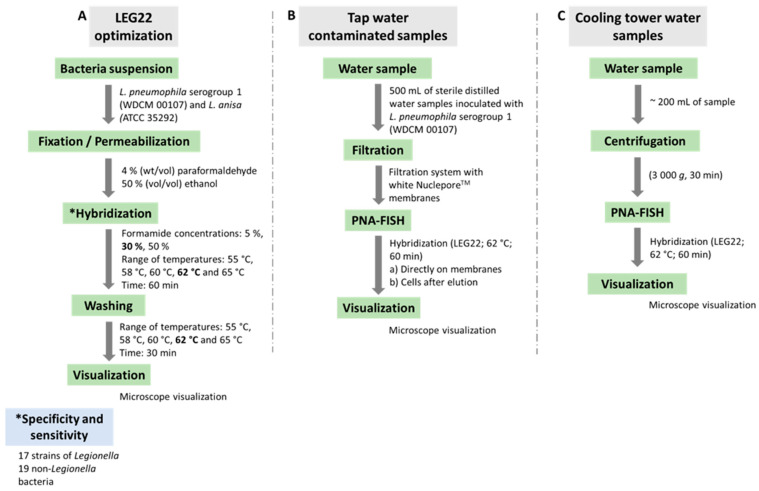
Representative diagram of the methodology used in the present study. (**A**) Optimization of LEG22 probe. Numbers in bold correspond to the selected parameters. Specificity and sensitivity were assessed subsequentely; (**B**,**C**) Application of PNA-FISH method in tap-water-contaminated samples and in cooling tower water samples, respectively.

Hybridization was performed as previously described by the group [13,35,36], with some modifications. As Figure 2A summarizes, suspensions of 2 × 10^7^ cell/mL of *L. pneumophila* serogroup 1 (WDCM 00107) and *L. anisa* ATCC 35,292 were dispensed in 8 mm well slides (Marienfeld, Lauda-Königshofen, Germany) and allowed to air dry. For permeabilization and fixation of *Legionella* spp. cells, 30 µL of 4% (wt/vol) paraformaldehyde followed by 50% (vol/vol) ethanol, for 10 min each, were dispensed in the wells at room temperature and allowed to air dry. The slides were then covered with 20 µL of hybridization solution containing 10% (wt/vol) dextran sulfate, 10 mM NaCl, 30% (vol/vol) formamide, 0.1% (wt/vol) sodium pyrophosphate, 0.2% (wt/vol) polyvinylpyrrolidone, 0.2% (wt/vol) Ficoll, 5 mM disodium EDTA, 0.1% (vol/vol) Triton X-100, 50 mM Tris-HCl (pH 7.5) (all from Sigma-Aldrich, Sintra, Portugal, except disodium EDTA, which was from Pronalab, Lisbon, Portugal) and 200 nM of the PNA probe (Eurogentec, Belgium). The slide wells were covered with coverslips, protected from the light, and incubated for 60 min at different temperatures under study. Following hybridization, the slides were transferred to a coupling jar containing prewarmed washing solution, containing 5 mM Tris base, 15 mM NaCl and 1% (vol/vol) Triton X (pH 10) (all from Sigma-Aldrich, Sintra, Portugal). The samples were allowed to air dry, mounted with a drop of nonfluorescent immersion oil and covered with coverslips.

Several temperatures (55 °C, 58 °C, 60 °C, 62 °C and 65 °C) and formamide concentrations (5%, 30% and 50%) (at temperatures 55 and 60 °C) were studied for signal-to-noise ratio assessment. Slides were stored in the dark for a maximum of 24 h before microscopy visualization. After optimization of the hybridization conditions, the probe was applied for the other *Legionella* and non-*Legionella* strains described in Table 1 to assess the specificity and sensitivity of the probe.

### 2.4. PNA-FISH Method Testing in Water Samples

The first phase of *Legionella* spp. detection methodology comprises a concentration of the water samples, usually by filtration or centrifugation [6,11]. To evaluate the PNA-FISH detection ability by filtration, 500 mL of sterile distilled water samples was inoculated with 1 mL of different concentrations of *L. pneumophila* serogroup 1 (WDCM 00107) (corresponding to a final concentration from 10^5^ to 10^−1^ CFU/L), using a standard filtration system (PALL, Show Low, AZ, USA) with white Nuclepore^TM^ (Whatman^TM^, Maidstone, UK) membranes with a diameter of 47 mm and a pore size of 0.22 µm. These concentrations correspond to a concentration range of approximately 10^8^ to 10^2^ CFU in the filtration membrane.

To optimize the PNA-FISH method after filtration, 2 protocols were used: direct detection and elution. For direct detection, after filtering the sample, the membranes were allowed to air dry and overlaid with 4% (wt/vol) paraformaldehyde followed by 50% (vol/vol) ethanol, for 10 min each, at room temperature. The membrane was then placed on a glass slide and allowed to air dry. For the hybridization, 60 µL of hybridization solution (pH 7.5) containing 200 nM of the PNA probe was spread within the membrane with the help of a coverslip and incubated with light refraining. Subsequently, the filters were removed gently from the slide, transferred into a Petri dish, also protected from light, and filled with a prewarmed washing solution (pH 10) and incubated for 30 min. The samples were then allowed to dry, mounted with a drop of nonfluorescent immersion oil and covered with coverslips. Slides were stored in the dark for a maximum of 24 h before microscopy visualization (Figure 2B).

For the elution test, in order to resuspend adherent cells from the membrane, after filtering the sample, the membranes were agitated (270 rpm) in 5 mL of sterile distilled water in a Falcon tube for 20 min at room temperature. Then, the entire volume of the sample was centrifuged at 15,000× *g* for 10 min, and the supernatant was removed. The sample was resuspended in 100 µL of sterile distilled water and placed on a microscopic slide and allowed to air dry. The PNA-FISH protocol was carried out as described above. To determine the number of CFU per sample in all the experiments, 100 µL of the appropriate dilutions was plated onto BCYE plates at 37 °C for 4 days.

To test the PNA-FISH implementation in real water samples, preliminary assays using 10 cooling tower water samples supplied by INSA-DSA ASMIP were also performed (Figure 2C). These samples were firstly tested by INSA-DSA ASMIP, using the culturing method based on the ISO 11731:2017 [6], and all were <10^3^ CFU/L, one of them negative. As these samples have more interferents, we performed a preliminary centrifugation step before applying the hybridization procedure, based on the ISO 11731:2017 [6]. Briefly, 200 mL of each sample was centrifuged at 3000× *g* for 30 min; then, the supernatant was carefully removed, leaving only 1 mL. Subsequently, the hybridization step was performed as described above.

### 2.5. Microscopy Visualization

Microscopy visualization was performed using a Nikon Eclipse 80i (Japan) epifluorescence microscope with a camera NikonDS-Fi1 (Izasa, Japan), equipped with a filter sensitive to the Alexa Fluor 488 molecule attached to the PNA probe (excitation, 482/35 nm; emission, 536/40 nm). Visualization was also assessed with the other filters present in the microscope that are not sensitive to the probe fluorescence signal. For every experiment, a negative control was performed simultaneously, for which all the steps described above were carried out, but no probe was added during the hybridization procedure. All the images were acquired using NIS-Elements B.R. 3.2 (Izasa, Japan) software with a magnification of ×100.

### 2.6. Statistical Analysis

The statistical validity of specificity and sensitivity parameters and respective 95% confidence intervals (CIs) were determined using the VassarStats: Website for Statistical Computation (http://vassarstats.net; assessed on June 2020 and June 2022).

## 3. Results

### 3.1. Probe Design

To make a first selection of possible regions for probe design, 16S rRNA gene sequences of *Legionella* were aligned with the closest relatives’ strains and with other bacteria that might be present in water. The selection was based on regions that showed differences between the *Legionella* sequences to the non-target strains. Hence, the selected probe, with the best compromise between the number of targets and non-targets detection, was N terminal-TCC ACT ACC CTC TCC-C terminal.

The sequence targeted the 16S rRNA between positions 634 and 648 of the *Legionella pneumophila* subsp. *pneumophila* JCM 7571 (Accession number AB594755; SILVA database). The probe was named LEG22, regarding the numbering of sequences tested in 16S rRNA regions analyzed.

The theoretical specificity and sensitivity of the probe were further evaluated using the Probe Match tool (RDP-II) program. The search confirmed that LEG22 had 99.9% (95% CI, 99.8–99.9) specificity and 96.9% (95% CI, 96.5–97.2) sensitivity. In order to compare the probe developed in this study with other *Legionella* spp. PNA probes found in the literature, the theoretical specificity and sensitivity of the probe PLEG200 [32] were also evaluated with the same software program (Table 2). The search showed that PLEG200 presented an acceptable level of specificity (100%; 95% CI, 99.8–99.9) but lower sensitivity (76.4%; 95% CI, 74.0–78.8).

### 3.2. PNA-FISH Perfomance

**Figure 3:**
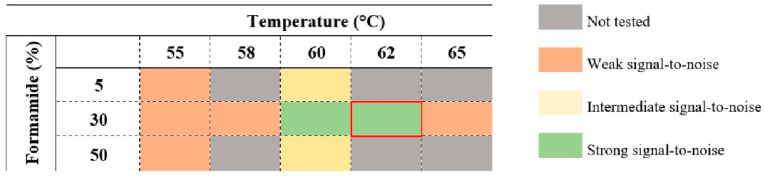
Temperature and formamide concentration assessments for hybridization conditions optimization. The selected conditions for the best performance are 62 °C and 30% formamide (red bracket).

**Figure 4:**
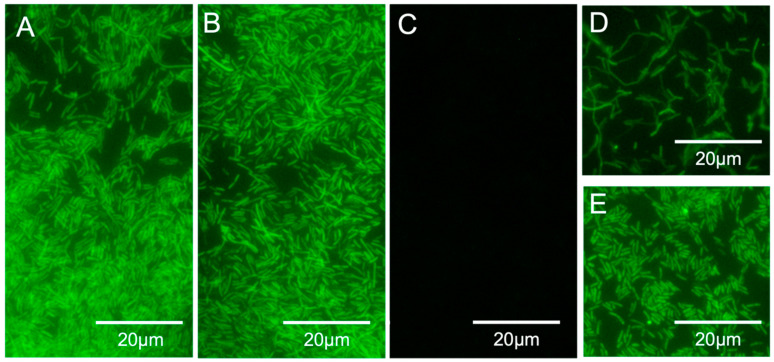
(**A**–**C**) PNA-FISH specificity and sensitivity results for the LEG22 peptide nucleic acid probe. (**A**) *Legionella pneumophila* serogroup 1, WDCM 00107; (**B**) *Legionella anisa,* ATCC 35292; (**C**) *Escherichia coli* (INSA isolate); (**D**,**E**) *L. pneumophila* serogroup 1, WDCM 00107 in filtered artificially contaminated water samples (10^8^ CFU per sample). (**D**) PNA-FISH on the membranes; (**E**) PNA-FISH on eluted cells. Images were obtained with equal exposure times.

Although the protocol is generally straightforward, some aspects of the hybridization conditions had to be optimized to evaluate the optimal conditions for the LEG22 PNA probe to work. To guarantee that the probe efficiently accesses and hybridizes with the target sequence, different formamide concentrations (5%, 30% and 50%) were evaluated at 55 °C and 60 °C (the range of temperatures that usually presents higher stringency). As the results were better at 30% formamide, we then tested different hybridization temperatures (between 55 °C and 65 °C) to adjust the best hybridization conditions. The best performance in terms of strongest signal-to-noise ratio was achieved at 62 °C with 30% formamide (Figure 3 and Figure 4).

Once the hybridization method was optimized, the specificity and sensitivity of the LEG22 PNA probe was tested. For this, the optimized protocol was applied to 17 *Legionella* and to 19 non-*Legionella* strains. As shown in Table 1 and Figure 4A–C, all *Legionella* strains were detected, whereas no hybridization was observed for the other species used. Therefore, 100% (95% CI, 77.1–100) specificity and 100% (95% CI, 79.1–100) sensitivity for the probe was obtained.

### 3.3. Detection in Water Samples

After probe optimization, we tested the method in the water filtration procedure using artificially contaminated tap water (from 10^8^ to 10^2^ CFU per sample) with *L. pneumophila*. We were able to detect the bacteria after filtration, both directly on the filter and by eluting the cells (Figure 4D,E and Table 3) above 10^1^ CFU/L.

Nevertheless, several cooling tower water samples provided by INSA-DSA ASMIP were tested directly with PNA-FISH. All the samples were tested by culture using ISO11731:2017 [6] (<10^3^ CFU/L, one negative). Nonetheless, no positive result was observed with PNA-FISH for all the samples, which demonstrates that there is still a need to further optimize the protocol in order to detect cells in more turbid samples, since signal interference was observed in some of the samples, as they presented more residues.

## 4. Discussion

The cases of LD reported have been rising worldwide in recent decades. In Portugal, significant LD outbreaks were identified between 2014 and 2020, with more than 550 confirmed cases and 30 deaths [37,38,39]. European guidelines for *Legionella* identification require the implementation of monitoring and water treatment actions for risk assessment [9]. As water systems are the most significant sources of *Legionella* infections, it is imperative from a public health perspective to survey these aquatic environments for the presence of these bacteria [7].

Notwithstanding, as stated above, the currently available approaches to routine do not fully comply with the requirements for *Legionella* testing. FISH can take a step forward as a powerful and rapid tool for identification of microbial populations without the need for time-consuming microorganisms culturing [13,40,41]. Additionally, PNA-FISH overcomes problems related to PCR-based molecular technologies, such as susceptibility to inhibitors, cross-contamination, false positive results and even the requirements for specialized personnel [24,42]. Critically, in addition to technical issues, PCR-based methods can be troublesome and require specific and expensive equipment (for nucleic acid extraction and amplification steps), limiting their use to centralized laboratories. PNA-FISH is a rapid method, as it takes approximately 3 h to provide the result, with a simple workflow, as it does not consist of a technically demanding protocol, and apart from the visualization equipment, it does not require any overpriced equipment. This work intends to give insights about the development of a PNA-FISH method for the detection of *Legionella* spp. in water.

Traditionally, probes target the 16S/23S rRNA sequence of the bacteria, since these structures are abundant and highly conserved in cells (e.g., 10^4^–10^5^ for *Escherichia coli*) [43,44]. PNA, as shorter and neutrally charged molecules, can confer extra efficiency to the reaction, as they can present improved affinity to the target, hence increasing specificity [19]. From the sequences analyzed in different regions of the 16S rRNA, LEG22 was the probe selected for having the best theoretical performance, presenting 99.9% specificity and 96.9% sensitivity. In order to compare the probe developed in this study with other potential probes developed earlier, we conducted an extensive literature search. To the best of our knowledge, only one *Legionella* spp. PNA probe previously published was found in the literature, PLEG200 [32]. Nevertheless, PLEG200 was based on an existing DNA probe sequence (LEG226), which targets a low-binding-affinity site in the 16S rRNA [15,32]. In that work, ribosomal databases analysis indicated that this probe only bound to the *Legionella* spp. sequences deposited. Here, and 15 years later, we evaluated this probe again, together with LEG22, with the Probe Match tool (RDP-II) (Table 2) (last accessed in October 2021). The search showed that both probes presented acceptable levels of specificity. However, regarding sensitivity, PLEG200 showed to be less sensitive (76.4%). This may be explained, as these databases are constantly being updated, new sequences being deposited over time, and these changes can affect the accuracy of the probes, since these parameters will be related to the quality and quantity of sequences available in the analysis period [19]. Nevertheless, in practical terms, the criterion for the probe’s choice will fall on the aim of the study. If the criterion is focused on physiology studies or on the inoculation of a specific bacteria in a biofilm system, the sensitivity is not so relevant, as long as the strain of interest is detected. Conversely, for the development of a diagnostic method for online monitoring and risk assessment, it is important to have very high sensitivity. Nonetheless, in the particular case of *Legionella*, as almost 100% of the bacteria that causes outbreaks is *L. pneumophila*, as long as the probes can detect this species, the problem should be less relevant. Nevertheless, both probes, PLEG200 [32] and the newly designed LEG22, were able to detect all the *Legionella* strains tested.

We carefully designed the LEG22 probe to target a conserved region in *Legionella* strains. In fact, no false negative or false positive results were found when we tested the LEG22 probe against *Legionella* spp. and non-*Legionella* spp. strains (Table 1), which corroborates the performance of this probe, providing a suitable alternative for studies in pure cultures but also in biofilms. The main advantage of using PNA rRNA probes is related with the visualization of the whole cells and spatial distribution and metabolic activity of the cells [45], allowing a better knowledge of the organization and functional development of biofilms.

In a FISH protocol, the variables temperature and formamide concentration must be well adjusted in order to optimize the hybridization performance [46]. Formamide destabilizes double-stranded molecules by interfering with hydrogen bonds [47,48], and therefore, reducing the hybridization temperature. It is expected that for each 1% (vol/vol) formamide added, the temperature decreases about 1 °C. It is common to test formamide concentrations ranging from 5% to 70% (vol/vol), depending on the type of microorganism, and to use a range of temperatures near 50–60 °C [46]. Here, we tested 55 and 60 °C with 5, 30 and 50% (vol/vol) formamide. Although the best condition was shown to be 30% formamide and 60 °C, we further tested different temperatures, and 62 °C was chosen as the final temperature, as it provided a better signal-to-noise result.

To assess if the method could be applied in environmental samples, tap water samples artificially contaminated with different *L. pneumophila* serogroup 1 (WDCM 00107) CFUs were used to adapt a filtration protocol based on ISO 11731:2017 [6]. For both protocols used (PNA-FISH directly on the membrane or after cells elution from the filter), the signal was suitable up to 10^1^ CFU/L with a good signal-to-noise ratio. For the less concentrated samples, detection with LEG22 was not possible, as the number of cells could have been below the detection limit of FISH using a microscope equipment. It was previously defined that for a trustworthy analysis, each microscopic field should contain at least five cells, which may not be the case in the 10^0^ and 10^−1^ CFU/L samples [15,49], even though this technique appears to be appropriate for the rapid detection of *Legionella* in contaminated water samples.

For samples with higher turbidity, further optimization of the method will be carried out to improve the detection. The cooling tower water samples were analyzed with an initial centrifugation protocol based on ISO 11731:2017 [6] due to the high presence of residues. The samples, although testing positive for culture (except for one sample) (<10^3^ CFU/L), were negative with PNA-FISH. One of the issues observed was the presence of autofluorescent water interferents that may hinder the detection of the cells’ fluorescent signal. In order to overcome the interference, the water samples pre-treatment will be a future concern. The implementation of a tangential flow filtration system to isolate and concentrate bacterial cells can effectively solve the problem [50]. However, compared to culture, which takes several days to provide results, PNA-FISH can be considered a rapid and reliable method for *Legionella* detection, as it takes only 3–4 h to perform the whole protocol.

FISH is a very versatile tool, and allied with microfluidic platforms, this method can, in the future, be implemented in routine monitoring practices in industrial settings.

## 5. Conclusions

This work describes the development of a new detection method for *Legionella* spp. in water, based on PNA-FISH. This method, using a LEG22 PNA probe, proved to be a very sensitive and specific method for *Legionella* spp. detection in pure cultures and in contaminated water samples. It was observed that for water samples with higher turbidity, coming from cooling towers, an improved filtration protocol is still needed. Nevertheless, the results showed that the PNA-FISH method remains a good alternative for rapid and accurate detection of *Legionella*.

## 6. Patents

A Portuguese patent was submitted, resulting from the work reported in this manuscript.

## Figures and Tables

**Table 1:** List of strains used in this study, together with the results obtained with the PNA-FISH probe specificity/sensitivity test. ATCC—American Type Culture Collection; WDCM—World Data Centre for Microorganisms.

Microorganisms	Origin	PNA-FISH Outcome
*L. anisa* * ^1^	ATCC 35292	+
*L. anisa* 144 ^1^	Treated Drinking Water	+
*L. bozemanii* ^1^	External Quality Assessment	+
*L. feeleii* 106 ^1^	Thermal Water	+
*L. feeleii* 187 ^1^	Cooling Tower Water	+
*L. feeleii* 242 ^1^	Irrigation Water	+
*L. feeleii* 263 ^1^	Treated Drinking Water	+
*L. geestiana* ^1^	Cooling Tower Water	+
*L. gormanii* ^1^	Thermal Water	+
*L. jamestowniensis* ^1^	External Quality Assessment	+
*L. jordanis* ^1^	External Quality Assessment	+
*L. longbeachae* ^1^	External Quality Assessment	+
*L. micdadei* 94 ^1^	External Quality Assessment	+
*L. micdadei* 99 ^1^	Untreated Drinking Water	+
*L. oakridgensis* ^1^	Thermal Water	+
*L. pneumophila* (serogroup 1) * ^1^	WDCM 00107	+
*L. pneumophila* (serogroup 2–15) ^1^	Treated Drinking Water	+
*Acinetobacter calcoaceticus* ^2^	Treated Drinking Water	−
*Aerococcus viridans* ^1^	External Quality Assessment	−
*Aeromonas hydrofila* ^1^	Environment	−
*Burkholderia cepacian* ^2^	Treated Drinking Water	−
*Enterobacter cloacae* ^1^	Environment	−
*Enterobacter aerogenes* ^1^	External Quality Assessment	−
*Enterococos faecalis* ^1^	External Quality Assessment	−
*Escherichia vulneris* ^1^	Environment	−
*Escherichia coli* ^1^	External Quality Assessment	−
*Klebsiella oxytoca* ^1^	External Quality Assessment	−
*Leifsonia aquatica* ^1^	External Quality Assessment	−
*Micrococcus luteus* ^1^	External Quality Assessment	−
*Pseudomonas fluorescens* ^1^	Environment	−
*Pseudomonas aeruginosa* ^1^	Environment	−
*Salmonella Wentworth* ^1^	External Quality Assessment	−
*Serratia liquefaciens* ^1^	Environment	−
*Staphylococcus warnerii* ^1^	Environment	−
*Staphylococcus aureus* ^1^	External Quality Assessment	−
*Stenotrophomonas maltophilia* ^2^	Treated Drinking Water	−

^1^ INSA-DSA ASMIP; ^2^ Simões et al. (2007) [30]; (+) Positive fluorescent result with PNA-FISH; (−) Negative fluorescent result with PNA-FISH; * Reference strain.

**Table 2:** Evaluation of the *Legionella* spp. PNA probes available.

Probe	Target Gene	Sequence (5′–3′)	Length (bp)	GC%	Specificity ^a^%	Sensitivity ^a^%	Position	Ref.
PLEG200	16S rRNA	GACGCAGGCTAATCT	15	53.3	100.0	76.4	226–240	[32]
LEG22	16S rRNA	TCCACTACCCTCTCC	15	60.0	99.9	96.9	634–648	This work

^a^ The theoretical determination of sensitivity and specificity was performed based on Almeida et al. (2010) [35] using the RDP-II database from the same period of time (September–October 2021).

**Table 3:** Artificially inoculated tap water results with PNA-FISH (both directly in the membrane and in the eluted cells) and with traditional culture.

Artificially Inoculated Tap Water		
CFU/L	PNA-FISH Outcome	Traditional Culture
10^5^	+	+
10^4^	+	+
10^3^	+	+
10^2^	+	+
10^1^	+	+
10^0^	−	+
10^−1^	−	+

(+) Positive result with PNA-FISH or traditional culture; (−) Negative result with PNA-FISH.
